# Chemical mimetics of the N-degron pathway alleviate systemic inflammation by activating mitophagy and immunometabolic remodeling

**DOI:** 10.1038/s12276-023-00929-x

**Published:** 2023-02-01

**Authors:** Prashanta Silwal, Young Jae Kim, Yoon Jee Lee, In Soo Kim, Sang Min Jeon, Taylor Roh, Jin Kyung Kim, Min Joung Lee, Jun Young Heo, Doo Sin Jo, Sang-Hee Lee, Dong-Hyung Cho, Jin Man Kim, Yong Tae Kwon, Eun-Kyeong Jo

**Affiliations:** 1 Department of Microbiology, Chungnam National University School of Medicine, Daejeon, 35015 Republic of Korea; 2 Infection Control Convergence Research Center, Chungnam National University School of Medicine, Daejeon, 35015 Republic of Korea; 3 Department of Medical Science, Chungnam National University School of Medicine, Daejeon, 35015 Republic of Korea; 4 Cellular Degradation Biology Center and Department of Biomedical Sciences, College of Medicine, Seoul National University, Seoul, 03080 Republic of Korea; 5 Department of Microbiology, Keimyung University School of Medicine, Daegu, Republic of Korea; 6 Department of Biochemistry, Chungnam National University School of Medicine, Daejeon, 35015 Republic of Korea; 7 School of Life Sciences, BK21 FOUR KNU Creative BioResearch Group, Kyungpook National University, Daegu, 41566 Republic of Korea; 8 Bio-Electron Microscopy Research Center (104 Dong), Korea Basic Science Institute, Ochang, Cheongju 28199 Republic of Korea; 9 Department of Pathology, Chungnam National University School of Medicine, Daejeon, 35015 Republic of Korea; 10 AUTOTAC Bio Inc., Changkyunggung-ro 254, Jongno-gu, Seoul, 03077 Republic of Korea; 11 SNU Dementia Research Center, College of Medicine, Seoul National University, Seoul, 03080 Republic of Korea; 12 Ischemic/Hypoxic Disease Institute and SNU Dementia Research Center, College of Medicine, Seoul National University, Seoul, 03080 Republic of Korea

**Keywords:** Sepsis, Mitophagy

## Abstract

The Arg/N-degron pathway, which is involved in the degradation of proteins bearing an N-terminal signal peptide, is connected to p62/SQSTM1-mediated autophagy. However, the impact of the molecular link between the N-degron and autophagy pathways is largely unknown in the context of systemic inflammation. Here, we show that chemical mimetics of the N-degron Nt-Arg pathway (p62 ligands) decreased mortality in sepsis and inhibited pathological inflammation by activating mitophagy and immunometabolic remodeling. The p62 ligands alleviated systemic inflammation in a mouse model of lipopolysaccharide (LPS)-induced septic shock and in the cecal ligation and puncture model of sepsis. In macrophages, the p62 ligand attenuated the production of proinflammatory cytokines and chemokines in response to various innate immune stimuli. Mechanistically, the p62 ligand augmented LPS-induced mitophagy and inhibited the production of mitochondrial reactive oxygen species in macrophages. The p62 ligand-mediated anti-inflammatory, antioxidative, and mitophagy-activating effects depended on p62. In parallel, the p62 ligand significantly downregulated the LPS-induced upregulation of aerobic glycolysis and lactate production. Together, our findings demonstrate that p62 ligands play a critical role in the regulation of inflammatory responses by orchestrating mitophagy and immunometabolic remodeling.

## Sepsis: chemical modulation of selective autophagy alleviates systemic inflammation

Sepsis is a life-threatening condition that occurs when the body’s response to infection damages its own organs. Autophagy protects the body by degrading harmful materials in the cell using the lysosome. It has been an outstanding question whether autophagy can fight systemic inflammation in sepsis. Researchers in South Korea led by Eun-Kyeong Jo at Chungnam National University and Yong Tae Kwon at Seoul National University developed small molecule chemicals that target the autophagic receptor p62/SQSTSM-1/Sequestosome-1 to induce therapeutic efficacy during septic responses. In a mouse model of septic shock, these orally administrative compounds halted the production of proinflammatory molecules and reduced oxidative stress in mitochondria by facilitating the autophagic turnover of damaged mitochondria. Chemical modulation of p62 is now emerging as a therapeutic strategy to treat sepsis responsible for 11 million deaths a year.

## Introduction

Sepsis with acute organ dysfunction is a life-threatening disease that affects more than 19 million people annually^1,2^. Sepsis is caused by a dysregulated immune response to harmful stressors and is recognized as a global health priority by the World Health Organization^3–5^. During sepsis, the uncontrolled production of inflammatory mediators in response to infectious and dangerous signals induces potentially harmful immune reactions that lead to organ dysfunction^3–5^. The uncontrolled production of proinflammatory cytokines such as interleukin (IL)-1β activates systemic inflammation associated with sepsis and septic shock. IL-1β synthesis is mediated by activation of the NOD-, LRR-, and pyrin domain-containing protein 3 (NLRP3) inflammasome complex^6–9^. Because autophagy activation counteracts the excessive inflammatory response, modulation of autophagy and/or mitophagy is a potential treatment for inflammatory diseases and sepsis^10–18^. In addition, immunometabolic remodeling is linked to the pathophysiological mechanisms that underlie sepsis^19,20^. Indeed, modulation of aerobic glycolysis in macrophages can protect mice from septic death and polymicrobial sepsis^19,20^. Therefore, it is crucial to identify novel therapeutic approaches for sepsis and septic shock based on the coordinated regulation of autophagy, inflammation, and immunometabolism.

Intracellular protein degradation is crucial for cellular biological functions and protein complex remodeling. N-terminal degron (N-degron) pathways trigger the degradation of short-lived target proteins with N-terminal signals, known as N-degrons^21–24^. Diverse routes to N-degrons have been reported, including protease-mediated cleavage of proteins to expose a destabilizing Nt-residue^25–27^ and enzymatic Nt-modifications such as Nt-arginylation and Nt-acetylation^22^. N-recognins are recognition components, such as specific UBR box E3 ligases and proteins that bind N-degrons^22,28^. Recent studies have revealed that the selective autophagy receptor p62/SQSTM1 functions as an N-recognin interacting with N-degrons such as the Nt-Arg of arginylated HSPA5/GRP78/BiP^29–31^. Furthermore, small-molecule synthetic ligands of the p62 ZZ domain, which is required for the p62 recognition of N-degrons, induce the autophagic degradation of destabilized protein aggregates^29–32^. Emerging evidence indicates that synthetic ligands of the ZZ domain of p62 (i.e., p62 ligands) have potential effects in alleviating various diseases associated with dysfunctional autophagy^32–34^. However, it is unknown whether p62 ligands have protective functions against pathological inflammatory responses in the context of sepsis and septic shock.

Here, we found that p62 ligands (ATB1021, YTK-2205, and ATB1071) attenuate inflammatory responses to lipopolysaccharide (LPS) in macrophages by activating mitophagy and immunometabolic remodeling. Treatment with p62 ligand alleviated systemic inflammation and decreased mortality in mice with LPS-induced septic shock and experimental sepsis. The p62 ligand significantly suppressed the production of interleukin (IL)-1β, IL-18, IL-6, and chemokines in response to multiple innate immune stimuli and NLRP3 inflammasome activation. Mechanistic analysis showed that the p62 ligand activated autophagy in unstimulated and resting macrophages. It also upregulated mitophagy via p62, thereby controlling mitochondrial dysfunction and the release of inflammatory cytokines. Moreover, the p62 ligand modulated aerobic glycolysis and lactate production in macrophages during inflammation. Collectively, these data demonstrate that p62 ligands can be novel potential therapeutics by activating mitophagy and immunometabolic remodeling, thereby mitigating systemic inflammation in response to various innate immune signals.

## Materials and methods

### Mice

Wild-type C57BL/6 mice were purchased from Samtako Bio (Osan, Gyeonggi-do, Korea) and maintained under specific pathogen-free conditions in the animal facilities at Chungnam National University School of Medicine. Mice were used in accordance with the guidelines of the Institutional Animal Care and Use Committee, Chungnam National University School of Medicine, Daejeon, Korea (approval nos. 202109A-CNU-180 and CNUH-021-A0011).

### Chemical synthesis of p62 ligands

ATB1021 (YT-6-2: (R)-1-(3,4-bis((4-fluorobenzyl)oxy)phenoxy)-3-((2-hydroxyethyl)amino)-propan-2-ol) was synthesized as previously described^35^. Details of the design and synthesis of YTK-2205 (2-((3,4-diphenethoxybenzyl)amino)ethan-1-ol), as well as its ability to modulate p62 in macroautophagy, are available from the World Intellectual Property Organization (https://patentscope.wipo.int/search/en/detail.jsf?docId=WO2019190172). Details of the design and synthesis of ATB1071 are available from the World Intellectual Property Organization (PCT/KR2021/017547).

### Mouse models

For the LPS-induced sepsis model, mice were intraperitoneally (IP) injected with LPS (28 mg/kg; Sigma‒Aldrich, St. Louis, MO, USA; L3755) and observed for survival at 12 h intervals; the overall survival rate was calculated. At 2 h before LPS injection (for the pretreatment model), mice were IP injected with vehicle control or ATB1021 (20 mg/kg; *n* = 4 per group). In a separate set of experiments, mice were IP injected with vehicle control or ATB1021/YTK-2205 (20 mg/kg) 2 h before and 1 h after (for pre- and posttreatment models) or ATB1021 (20 mg/kg) 1 h after (for the posttreatment model) LPS injection (*n* = 6–8 per group). To harvest organs, ATB1021 (20 mg/kg)- or vehicle-treated mice were euthanized 6 h after LPS injection (14 mg/kg, IP, *n* = 6 per group). Lung and spleen tissues were homogenized and then used for RNA preparation and enzyme-linked immunosorbent assays (ELISAs). Lung tissue was harvested for histopathological analysis and staining.

For the cecal ligation and puncture (CLP) model of sepsis, mice (*n* = 7 per group) were anesthetized and subjected to abdominal incision. The cecum was exposed, ligated, and punctured twice with a 21 gauge needle. It was then returned to the abdomen, and the peritoneum and skin were closed with sutures. Animals were IP injected with vehicle control or ATB1021 (20 mg/kg) 2 h before and 12 h after the CLP procedure. Mice were observed for survival, and the overall survival rate was calculated.

For the polyinosinic-polycytidylic acid (poly(I:C))-induced acute lung injury (ALI) mouse model, 10 mg/kg poly(I:C) (Sigma‒Aldrich; P0913) was intranasally instilled. Vehicle control or ATB1021 (20 mg/kg) was IP injected 2 h before and 1 h after poly(I:C) instillation (*n* = 6 per group). At 6 h after poly(I:C) instillation, mice were euthanized to harvest lung tissue for RNA preparation and histological analysis.

### Cell preparation and treatment

Primary bone marrow-derived macrophages (BMDMs) and peritoneal macrophages (PMs) were cultured in Dulbecco’s modified Eagle’s medium (DMEM; Lonza, Basel, Switzerland; 12–604 F) plus fetal bovine serum (10% v/v; Gibco, Waltham, MA, USA; 16000–044) and penicillin‒streptomycin-amphotericin B (Lonza; 17–745E). Bone marrow cells were cultured for 5–7 days in DMEM containing 25 ng/mL macrophage colony-stimulating factor (R&D Systems, Minneapolis, MN, USA; 416-ML) to generate fully differentiated BMDMs.

PMs were prepared as previously described^36^. Briefly, 3 days after IP injection of 3% thioglycolate, mice were euthanized and IP injected with ice-cold phosphate buffered saline (PBS) containing 3% fetal bovine serum (5 mL). The wash solution was collected and centrifuged (1500 rpm, 8 min, 4 °C) to obtain a macrophage pellet. Cells were suspended in culture medium, counted, and seeded in plates for further analysis.

Before experiments, cells were incubated overnight in resting medium (DMEM with 5% fetal bovine serum). Cells were pretreated with vehicle, ATB1021, YTK-2205 or ATB1071 for 1 h and then stimulated with 100 ng/mL LPS (InvivoGen, San Diego, CA; tlrl-eblps), 20 μg/mL poly(I:C) (Sigma‒Aldrich), 10 μg/mL zymosan (Invivogen; tlrl; zyn), or 100 ng/mL Pam3CSK4 (InvivoGen; tlrl-pms) for 3, 6, or 18 h. Supernatants were collected for ELISAs; cells were lysed for RNA preparation or Western blotting. For autophagic flux analysis, bafilomycin A1 (BafA1) (Sigma‒Aldrich, b1793) was preincubated with the cells for 1.5 h at a final concentration of 100 nM.

### RNA preparation and real-time polymerase chain reaction

TRIzol reagent (Invitrogen, Waltham, MA, USA; 15596026) was used for total RNA extraction; Reverse Transcriptase Premix (ELPIS Biotech, Daejeon, Korea; EBT-1515) was used for cDNA synthesis following the manufacturer’s protocol. Real-time polymerase chain reaction (PCR) was performed using SYBR Green Master Mix (Qiagen, Germantown, MD, USA; 204074) in the Rotor-Gene Q 2plex system (Qiagen). The 2^ΔΔ^ threshold cycle (C_t_) method with normalization to β-actin or glyceraldehyde-3-phosphate dehydrogenase was used to analyze and express the data in terms of relative fold changes. Primer sequences are listed in Supplementary Table 1.

### Western blotting

Cells were washed with cold PBS and then lysed in radioimmunoprecipitation assay buffer supplemented with protease inhibitor cocktail (Roche, Basel, Switzerland; 11836153001) and phosphatase inhibitor cocktail (Sigma‒Aldrich; P5726) to obtain protein samples. Equal amounts of proteins were mixed with sodium dodecyl sulfate sample buffer (ELPIS Biotech, Daejeon, Korea; EBA-1052) and boiled for 7 min. Denatured protein samples were then subjected to sodium dodecyl sulfate–polyacrylamide gel electrophoresis. The separated proteins were transferred to polyvinylidene difluoride membranes (Millipore, Billerica, MA, USA; IPVH0001). The membranes were incubated at room temperature for 1 h in Tris-buffered saline containing 0.1% Tween 20 (TBS-T) with 5% skim milk. Blocked membranes were incubated overnight with primary antibodies at 4 °C. Next, the membranes were washed with TBS-T and incubated with the corresponding secondary antibodies for 1 h at room temperature. After several washes with TBS-T, the results were visualized using enhanced chemiluminescence reagent (Millipore; WBKLS0500) in a UVitec Alliance mini-chemiluminescence device (UVitec, Cambridge, UK). The following primary antibodies were used: anti-phospho-nuclear factor (NF)-κB p65 (Ser536) (3033, 1:2000), anti-phospho-stress-activated protein kinase/Jun-amino-terminal kinase (SAPK/JNK; 4668, 1:1000), anti-p44/42 mitogen-activated protein kinase (MAPK) Erk1/2 (9101, 1:2000), and anti-phospho-p38 (4511, 1:2000) (all from Cell Signaling Technology [Danvers, MA, USA]); anti-beta-actin (sc-47778, 1:4000; Santa Cruz Biotechnology [Dallas, TX, USA]); anti-IL-1 beta (ab2105, 1:2000; Abcam [Cambridge, UK]); anti-LC3 (L8918, 1:1000; Sigma‒Aldrich); and anti-hypoxia-inducible factor-1 (HIF-1) alpha (NB100–479, 1:1000; Novus Biologicals [Centennial, CO USA]).

### Inflammasome analysis

PMs were seeded in 24-well plates and sensitized with LPS (100 ng/mL) in Opti-MEM for 4 h and then stimulated with 5 mM adenosine triphosphate (ATP; Sigma‒Aldrich; A5394) for 45 min. ATB1021 (10 μM) was added 1 h before ATP stimulation. Plates were placed on ice to stop the stimulation; supernatants were collected and centrifuged to remove debris. Samples were stored at −80 °C for ELISAs or further processed for protein precipitation. Cell lysates were prepared in radioimmunoprecipitation assay buffer for Western blotting. Proteins were precipitated from the supernatant using StrataClean Resin (Agilent Technologies, La Jolla, CA, USA; 400724). To each sample (500 μL), 5 μL of StrataClean Resin was added, vortexed for 1 min, and centrifuged at 210×*g* for 2 min at 4 °C. The supernatant was discarded; the pellet was resuspended in 1× sodium dodecyl sulfate sample buffer, boiled for 7 min, and subjected to western blotting.

### ELISA

Cell supernatants or lysates from in vivo samples were stored at −80 °C before analysis using the following kits: BD OptEIA Set ELISA Kit for IL-6 (555240) from BD Biosciences (San Diego, CA, USA) and IL-1B ELISA Kit (88–7013–88) from Invitrogen. ELISAs and data analysis were performed following the manufacturers’ protocols.

### Histology

Lung tissues were fixed in 10% formalin and embedded in paraffin wax. Four-micrometer-thick sections were prepared and then stained with hematoxylin and eosin (H&E). Whole fields of lung tissue were scanned; inflamed areas were quantified after determination of the mean fluorescence intensity in the red channel using FIJI software.

### Measurement of mitochondrial reactive oxygen species

Macrophages grown on coverslips were pretreated with vehicle, 10 μM ATB1021, or 200 μM MitoTEMPO (Sigma‒Aldrich; SML0737) and then stimulated with 100 ng/mL LPS for 30 min or 2 h; mitochondrial superoxide staining was performed with 2.5 μM MitoSOX Red (Invitrogen; M36008) for 30 min. Cells were washed with PBS, fixed in 4% paraformaldehyde (PFA) for 15 min, and mounted with Fluoromount-G^TM^ Mounting Medium with 4′,6-diamidino-2-phenylindole (DAPI; Invitrogen; 00–4959–52). After mounting, the cells were visualized using a confocal laser-scanning microscope.

### Assessment of mitophagy by mtDNA measurement

To determine mtDNA copy number, total DNA was extracted using a DNeasy Blood and Tissue Kit (Qiagen, Germantown, MD, USA; 69504) from PMs. Total DNA was consumed for real-time PCR using SYBR Green Master Mix (Qiagen; 204074) in the Rotor-Gene Q 2plex system (Qiagen). The 2^ΔΔ^ threshold cycle (Ct) method with normalization to that of the nuclear reference gene HK2 was used to analyze and express the data in terms of relative fold changes. The primer sequences (5 to 3 primers) for mtDNA were as follows: 16 S rRNA (mtDNA) forward, 5-CCG CAA GGG AAA GAT GAA AGA C-3 and reverse, 5-TCG TTT GGT TTC GGG GTT TC-3; ND1 (mtDNA) forward, 5-CTA GCA GAA ACA AAC CGG GC-3 and reverse, 5-CCG GCT GCG TAT TCT ACG TT-3; and hexokinase (nuclear DNA) forward, 5-GCC AGC CTC TCC TGA TTT TAG TGT-3 and reverse, 5-GGG AAC ACA AAA GAC CTC TTC TGG-3.

### Immunofluorescence staining and confocal microscopic analysis

For the immunofluorescence staining of autophagic analysis, the cells were fixed in 4% PFA for 15 min at 37 °C and permeabilized with 0.25% (v/v) Triton X-100 (Sigma‒Aldrich, T8787) in PBS for 20 min at room temperature. Cells were then stained with anti-LC3 pAb (1:400; MBL CO., LTD., Tokyo, Japan; PM036) or anti-LAMP1 mAb (1:400; Santa Cruz Biotechnology, sc-19992) to analyze autophagy. For mitophagic analysis, cells were stained with 100 nM MitoTracker Deep Red (Thermo Fisher Scientific, Waltham, MA, USA; M22426) in prewarmed DMEM (Lonza; 12–604 F) for 30 min at 37 °C, fixed in 4% PFA, permeabilized with 0.25% (v/v) Triton X-100 (Sigma‒Aldrich, T8787), and stained with anti-LC3 pAb or anti-LAMP1 mAb. Cells were washed with PBS and then incubated with secondary antibodies (for LC3 staining, Alexa Fluor 488-conjugated anti-rabbit IgG [Thermo Fisher Scientific; A11006], 1:400; for LAMP1 staining, Alexa Fluor 488-conjugated anti-rat IgG [Thermo Fisher Scientific; A11008], 1:400) for 1 h. The cells were then mounted with Fluoromount-G^TM^ mounting medium with DAPI. For neutrophil staining, 3-μm-thick lung sections were prepared and stained with the primary anti-mouse Ly6G antibody (Bio X Cell, Lebanon, NH, USA; BP0075–1), and the nuclei were stained with DAPI (Invitrogen, P36935).

After mounting, images were visualized and captured using a TCS SP8 confocal laser-scanning microscope (Leica) and accompanying software (LAS X Small 2.0; Leica). Image capture parameters such as excitation, emission, and exposure time were kept constant. Each condition was assayed in quadruplicate, and at least 100 cells per field were counted. FIJI software was used to quantify LC3 punctate dots, LAMP1 intensities, or colocalization analysis in images by calculating Pearson’s correlation coefficient or EzColocalization.

### Transmission electron microscopy

Cells were scraped from plates and pelleted by centrifugation. Samples were sequentially fixed in 3% glutaraldehyde and 1% osmium tetroxide on ice for 1 h, washed with 0.1 M cacodylate buffer (pH 7.2) containing 0.1% CaCl_2_ and dehydrated in a series of ethanol and propylene oxide solutions. Next, samples were embedded in an Epon 812 mixture and polymerized in an oven at 60 °C for 36 h. Using an ULTRACUT UC7 ultramicrotome (Leica, Austria), ultrathin sections of 70 nm thickness were cut and mounted on 75-mesh copper grids. Sections were counterstained with uranyl acetate for 10 min and lead citrate for 7 min and then examined with a KBSI Bio-High Voltage EM (JEM-1400 Plus at 120 kV and JEM-1000BEF at 1000 kV; JEOL Ltd., Tokyo, Japan).

### Lactate measurement

Supernatants from LPS-stimulated PMs cultured in the presence or absence of ATB1021 were collected and filtered using Amicon Ultra 10 K Centrifugal Filters (Millipore; UFC501096). Lactate levels in the supernatants were assayed using the Lactate Colorimetric/Fluorometric Assay Kit (BioVision, Waltham, MA, USA; K607–100) according to the manufacturer’s instructions.

### Extracellular acidification rate analysis

Extracellular acidification rate (ECAR) measurements were performed using a Seahorse Bioscience XF24 Analyzer (Agilent Technologies). PMs were seeded at 2.5 × 10^5^ cells per well in an XF24 cell culture microplate, incubated overnight at 37 °C, and treated with vehicle or ATB1021 (10 μM) for 1 h before stimulation with LPS (100 ng/mL) for 6 h. Before analysis, the medium in each well was exchanged for 590 µL of assay medium (XF base medium containing 1 mM l-glutamine [pH 7.4]), and the plate was incubated in a non-CO_2_ incubator for 1 h at 37 °C. The XF24 Biosensor Cartridge was activated for 24 h in XF calibrant solution (1 mL/well, Agilent Technologies) at 37 °C in a non-CO_2_ incubator. The basal ECAR was measured in glycolysis stress test medium, and sequential injections of the following reagents were performed: 100 mM glucose (to fuel glycolysis, final concentration 10 mM), 100 μM oligomycin (to inhibit ATP synthase in mitochondria, final concentration 10 μM), and 500 mM 2-deoxyglucose (a competitive inhibitor of glucose, final concentration 50 mM). All steps were performed at 37 °C, and the data are presented relative to the total number of cells.

### Production and transduction of mouse p62 lentiviral short hairpin RNA

Short hairpin RNA (shRNA) was produced using pLKO.1-based target shRNA plasmids (m*p62*, TRCN0000238133) from Sigma‒Aldrich and the appropriate packing plasmid, as in our previous work^35^. For lentiviral infection, PMs or BMDMs cultured in 48- or 24-well plates were infected with lentiviral vector at a multiplicity of infection of 10 for 36 h and then treated with ATB1021 (10 μM) and LPS (100 ng/mL) for the indicated times.

### RNA sequencing and data analysis

For RNA sequencing, total RNA was isolated using TRIzol reagent (Invitrogen) following the manufacturer’s instructions. RNA quality was checked using a 2100 Bioanalyzer System (Agilent Technologies, Amstelveen, Netherlands), and RNA was quantified using a NanoDrop™ 2000 Spectrophotometer (Thermo Fisher). For cDNA library construction, the QuantSeq 3′-mRNA-Seq Library Prep Kit (Lexogen, Wien, Austria) was used per the manufacturer’s protocol. Briefly, 500 ng of total RNA from each sample was hybridized to oligo(dT) primers, and reverse transcription was carried out. The oligo(dT) primer sequence was Illumina-compatible at the 5’ end. After RNA template degradation, second-strand synthesis was initiated using a random primer that contained a linker sequence with Illumina compatibility at the 5′-end. Magnetic beads were used to remove reaction components before amplification of the double-stranded library to add all adapter sequences essential for cluster formation. After library purification, the NextSeq 500 instrument was used for high-throughput sequencing (single-read, 1 × 75 bp; Illumina, CA, USA).

For data analysis, raw reads were quality checked with BBduk (https://sourceforge.net/projects/bbmap) to remove low-quality bases (<Q20). The mouse MM10 reference genome was used to map the remaining reads with Bowtie2 software^37^. Bedtools software was used to calculate gene read counts; EdgeR software was used to perform quantile normalization. Heatmaps of differentially expressed genes between groups were generated using the heatmap package (v. 1.0.12) in R software (v. 4.0.4).

### Statistical analysis

Data were analyzed using GraphPad Prism software (v. 5.0, 8.4.0, or 8.4.3). Two-tailed Student’s *t* tests, analyses of variance, or the Mann‒Whitney *U* test were applied for statistical comparisons. For in vivo survival experiments, the log-rank (Mantel‒Cox) test was used. Data are means ± standard errors of the means (SEM) or standard deviation (SD). Asterisks indicate the *P* values: *p < 0.05, **p < 0.01 and ***p < 0.001.

## Results

### p62 ligands alleviate systemic inflammation and mortality in mouse models of septic shock and experimental sepsis

**Fig. 1:**
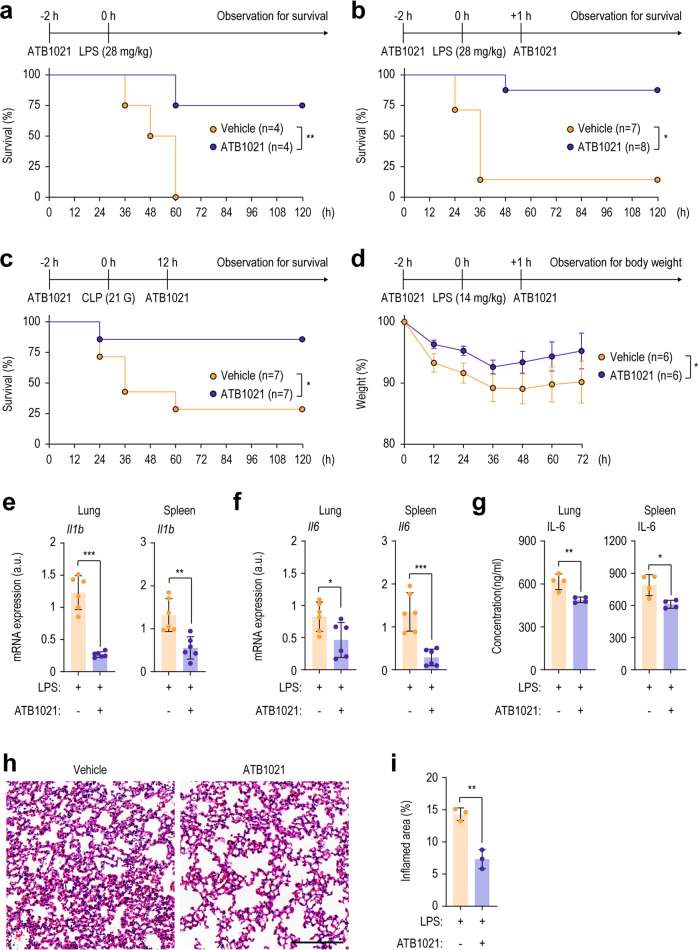
A p62 ligand prevents systemic inflammation and mortality in vivo. **a**, **b** Survival of mice for 120 h after injection with LPS (28 mg/kg) and pretreatment for 2 h with ATB1021 (20 mg/kg) (**a**, *n* = 4) or pre- and posttreatment for 2 and 1 h, respectively, with ATB1021 (20 mg/kg) (**b**, *n* = 7–8). Flowcharts of the experiments are shown. **c** Survival of control or ATB1021-treated mice 120 h after CLP (*n* = 7). Flowcharts of the experiments are shown. **d** Body weight observed for 72 h in vehicle- or ATB1021-treated and LPS-injected mice (*n* = 6). **e**–**g** Quantitative PCR analysis of *Il1b* (**e**) and *Il6* (**f**) mRNA levels and the IL-6 protein level (**g**) in lung and spleen tissues from mice treated with vehicle or ATB1021 for 2 h and injected with LPS (14 mg/kg) for 6 h (*n* = 4–6). **h**, **i** H&E-stained images (**h**) and inflamed areas (**i**, *n* = 3) in the lungs of mice treated with vehicle or ATB1021 (20 mg/kg) and injected with LPS (14 mg/kg). Flowcharts of the experiments are shown with means ± SEM (**e**–**g**, **i**). The log-rank (Mantel‒Cox) test (**a**–**c**), Mann‒Whitney *U* test (**e**, **f**, **i**), or two-tailed Student’s *t* test (**g**) was used to determine statistical significance. LPS lipopolysaccharide, CLP cecal ligation and puncture. *p < 0.05, **p < 0.01, ***p < 0.001.

To evaluate the effects of the p62 ligands on LPS-induced septic shock, we measured mouse survival rates after intraperitoneal challenge with LPS (28 mg/kg) and treatment with ATB1021 2 h before LPS (Fig. 1a), 1 h after LPS (Supplementary Fig. 1a), or 2 h before and 1 h after LPS administration (Fig. 1b). In all treatment regimens, the survival rate was significantly higher in ATB1021-treated mice than in vehicle-treated mice (*p* < 0.01 *vs*. vehicle; Fig. 1a or *p* < 0.05 *vs*. vehicle; Fig. 1b and Supplementary Fig. 1a). Mice treated with YTK-2205, another p62 ligand, also showed significantly higher survival than vehicle-treated mice (*p* < 0.01 *vs*. vehicle; Supplementary Fig. 1b). In mice with CLP-induced sepsis, the survival rate was significantly increased by ATB1021 (*p* < 0.05 *vs*. vehicle; Fig. 1c). Furthermore, ATB1021 prevented the loss of body weight for 72 h after LPS injection (*p* < 0.05 *vs*. vehicle; Fig. 1d).

To examine whether the increased survival was associated with decreased inflammatory responses in vivo, we evaluated cytokine levels in lung and spleen tissues at 6 h after LPS-induced septic shock. The mRNA and protein levels of IL-1β and IL-6 were significantly decreased in lung and spleen tissues from ATB1021-treated mice with sepsis compared to vehicle-treated mice (Fig. 1e–g). In addition, the mRNA expression levels of tumor necrosis factor (TNF)-α were significantly downregulated in lung and spleen tissues from ATB1021-treated mice compared to those from vehicle-treated mice at 1 and 2 h after LPS challenge (Supplementary Fig. 1c, d). H&E and anti-Ly6G antibody staining of lung tissues showed that LPS-induced histopathological damage and increased neutrophil infiltration (Ly6G^+^ area) in the vehicle-treated group were substantially attenuated in ATB1021-treated mice (Fig. 1h, i for H&E and Supplementary Fig. 1e, f for neutrophil infiltration). Therefore, ATB1021 treatment alleviates systemic inflammation and is protective against septic shock and experimental sepsis in mouse models.

### p62 ligands attenuate the production of proinflammatory cytokines in response to LPS and NLRP3 inflammasome stimuli

**Fig. 2:**
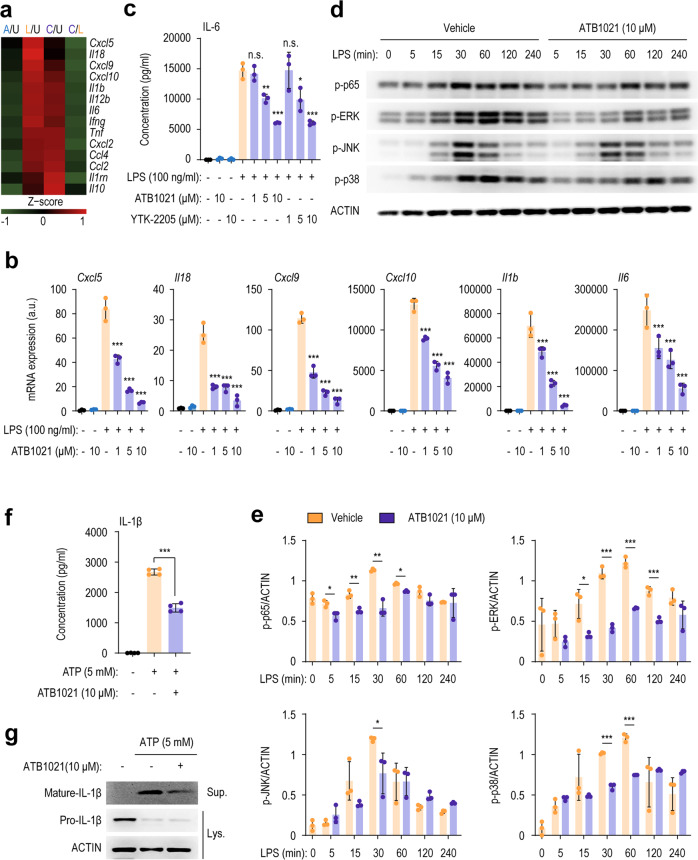
p62 ligands inhibit LPS-induced proinflammatory cytokine production and inflammasome activation. **a** Heatmap of differentially expressed genes in LPS-stimulated macrophages in the presence or absence of ATB1021 (10 μM). *Z* scores were calculated using the mean values of biological replicates. A/U, ATB1021 relative to unstimulated; L/U, LPS relative to unstimulated; C/U, ATB1021 plus LPS relative to unstimulated; C/L, ATB1021 plus LPS relative to the LPS stimulated group. **b** mRNA levels of *Cxcl5*, *Il18*, *Cxcl9, Cxcl10*, *Il1b*, and *Il6* in PMs pretreated with ATB1021 (1, 5, or 10 μM) for 1 h and stimulated with LPS (100 ng/mL) for 6 h (*n* = 3). **c** ELISA of supernatant collected 18 h after LPS (100 ng/mL) stimulation of PMs treated with ATB1021 or YTK-2205 (1, 5, or 10 μM) (*n* = 3). **d**, **e** PMs pretreated with ATB1021 (10 μM) were stimulated with LPS (100 ng/mL) for the indicated times to assess the phosphorylation of NF-κB and the MAPKs ERK, JNK, and p38. **e** Densitometry analysis of the indicated proteins (*n* = 3). **f**, **g** IL-1β levels in the culture supernatants of PMs primed with LPS (100 ng/mL) and stimulated with ATP (5 mM) in the presence or absence of ATB1021 (10 μM) (as determined by ELISA; **f**, *n* = 4) or in the supernatant and cell lysate (as determined by Western blotting; **g**) for mature or precursor IL-1β. Means ± SD are shown (**b**, **c**, **e**, **f**). One-way ANOVA with Tukey’s multiple comparison test (**b**, **c**) or two-tailed Student’s *t* test (**c**, **f**) was used to determine statistical significance. *p < 0.05, **p < 0.01, ***p < 0.001.

Because the p62 ligands attenuated systemic inflammation, we assessed their effects on proinflammatory responses in macrophages, the principal cells of antimicrobial defense and inflammatory responses^38^. RNA-seq analysis showed that the LPS-induced inflammation-related gene levels in BMDMs were significantly downregulated by ATB1021 (Fig. 2a, heatmap). It was noted that LPS-induced expression levels of multiple proinflammatory mediators, including C-X-C motif chemokine ligand 5 (*Cxcl5*), *Il18, Cxcl9*, *Cxcl10, Il1b*, *Il6*, chemokine ligand 2 (*Ccl2)*, and *Ccl4*, were markedly downregulated by treatment with ATB1021 (Fig. 2a). Validation by quantitative reverse transcription (RT)-PCR confirmed that the LPS-induced mRNA levels of *Cxcl5*, *Il18*, *Cxcl9*, *Cxcl10, Il1b* and *Il6* were downregulated in PMs by ATB1021 in a dose-dependent manner (Fig. 2b). In addition, LPS-induced expression of *Cxcl2* and *Ccl2* was considerably downregulated in PMs by ATB1021 (Supplementary Fig. 2a). Similar to PMs, ATB1021 inhibited LPS-induced *Il1b* and *Il6* expression in BMDMs (Supplementary Fig. 2b). Furthermore, either ATB1021 or YTK-2205 treatment significantly inhibited LPS-induced IL-6 production in PMs (Fig. 2c) and BMDMs (Supplementary Fig. 2c). However, LPS-induced *Tnf* expression was marginally reduced or unchanged by ATB1021 in BMDMs (Supplementary Fig. 2d). Additionally, another p62 ligand, ATB1071, had an inhibitory effect on LPS-induced *Il1b* expression in PMs (Supplementary Fig. 2e).

Next, we examined the molecular mechanism by which p62 ligand modulates proinflammatory cytokine/chemokine production in macrophages. We thus analyzed LPS-induced intracellular signaling pathway activation in PMs in the presence or absence of ATB1021. ATB1021 suppressed the LPS-induced phosphorylation of NF-κB (p65) and the mitogen-activated protein kinases (MAPKs) ERK1/2; it also had small but statistically significant effects on the phosphorylation of the MAPKs JNK and p38 (Fig. 2d, e). Moreover, ATB1021 significantly inhibited the production (Fig. 2f) and maturation (Fig. 2g) of IL-1β, which were induced by stimulation of the NLRP3 inflammasome (i.e., LPS plus ATP). Therefore, the p62 ligands dramatically inhibited proinflammatory cytokine/chemokine production by macrophages stimulated with LPS or by NLRP3 inflammasome activation.

### p62 ligand attenuates the production of proinflammatory cytokines in response to innate immune stimuli

**Fig. 3:**
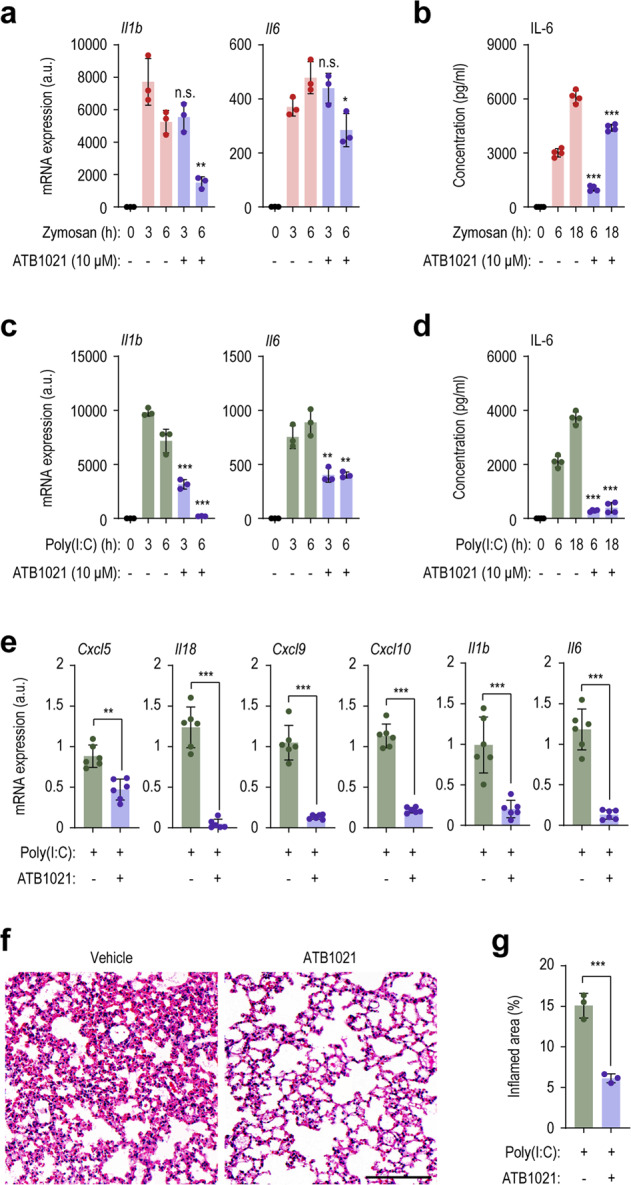
A p62 ligand exerts inhibitory effects against various innate stimuli. **a**–**d** PMs were pretreated with ATB1021 (10 μM) for 1 h and stimulated with zymosan (10 μg/mL; **a**, **b**) or poly(I:C) (10 μg/mL; **c**, **d**) for the indicated times; they were then analyzed by quantitative RT‒PCR to determine the mRNA levels of *Il1b* and *Il6* (**a**, **c**; *n* = 3) or by ELISA to determine the protein level of IL-6 (**b**, **d**; *n* = 4). **e** mRNA levels of *Cxcl5*, *Il18*, *Cxcl9*, *Cxcl10*, *Il1b* and *Il6* in lung tissues from mice instilled with poly(I:C) (10 mg/kg, intranasally) and treated with vehicle or ATB1021 (20 mg/kg; intraperitoneal) (*n* = 6). **f**, **g** H&E staining (**f**) and inflamed areas (**g**, *n* = 3) in the lungs of the mice treated as in **e**. Means ± SDs (**a**–**d**) and ±SEM are shown (**e**, **g**). Statistical analysis was performed by two-tailed Student’s *t* test (**a**–**d**) or the Mann‒Whitney *U* test (**e**, **g**). n.s., not significant, *p < 0.05, **p < 0.01, ***p < 0.001.

Given the findings that the p62 ligand ATB1021 attenuates LPS-induced inflammation, we evaluated whether the p62 ligand could suppress the inflammatory responses triggered by various innate immune stimuli. ATB1021 significantly decreased the *Il1b* and *Il6* mRNA levels in PMs in response to zymosan (Fig. 3a, an agonist of toll-like receptor 2 (TLR2) and Dectin-1), poly(I:C) (Fig. 3c, TLR3 agonist), and Pam3CSK4 (Supplementary Fig. 3a, TLR2/1 agonist). Consistent with these findings, the IL-6 levels in PM culture supernatants were significantly downregulated by ATB1021 (Fig. 3b, d and Supplementary Fig. 3b).

Next, we established a poly(I:C)-induced lung inflammation mouse model to assess the in vivo effect of ATB1021 on ALI, a mimic of inflammation caused by respiratory viral infections. Compared with vehicle-treated control mice, ATB1021-treated mice had reduced levels of the proinflammatory cytokines/chemokines *Cxcl5, Il18, Cxcl9*, *Cxcl10, Il1b*, and *Il6* (Fig. 3e) and *Ccl2* (Supplementary Fig. 3c) in the lung tissues after challenge with poly(I:C)-induced ALI. H&E staining of lung tissue showed that ATB1021 alleviated histopathological damage (Fig. 3f, g). These data indicate that the p62 ligand ATB1021 suppresses inflammatory responses triggered by various types of innate immune activation.

### A p62 ligand augments autophagy in LPS-stimulated macrophages

**Fig. 4:**
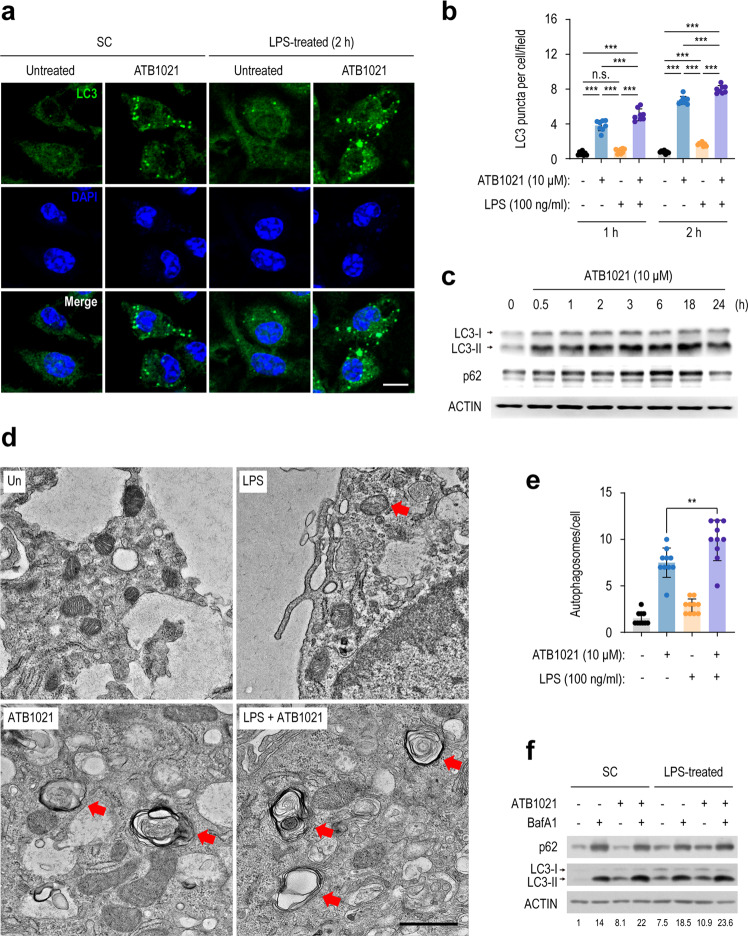
A p62 ligand induces autophagy in resting or LPS-stimulated macrophages. **a** PMs were treated with vehicle or ATB1021 (10 μM) for 1 h and stimulated with LPS (100 ng/mL) for 2 h; they were then immunostained using anti-LC3 antibody (green) and DAPI (nuclei, blue). Merged images are shown. Scale bar, 2 μm. **b** Number of LC3 puncta per cell in the images in **a**. **c** Western blotting analysis of LC3 lipidation in macrophages treated with ATB1021 (10 μM). Actin was used as the loading control. **d**, **e** Representative electron micrographs of PMs treated with vehicle or ATB1021 (10 μM) for 1 h and stimulated with LPS for 18 h. Red arrows indicate autophagosomes. Scale bar, 1 μm (**d**). Number of autophagosomes per cell (**e**). **f** RAW264.7 cells were treated with LPS (100 ng/mL) and ATB1021 (10 μM) for 9 h. Bafilomycin A1 (100 nM) was added 1.5 h before harvesting the cells to analyze autophagic flux. The LC3-II to LC3-I ratio is shown (bottom). Means ± SDs are shown (**b**, **e**). One-way ANOVA with Tukey’s multiple comparison test (**b**, **e**) was used to determine statistical significance. BafA1, bafilomycin A1; n.s., not significant, *p < 0.05, **p < 0.01, ***p < 0.001.

We previously found that p62 ligands are activators of autophagy and xenophagy^35^. Thus, we first examined whether ATB1021 activates autophagy in the presence or absence of LPS in macrophages. As expected, ATB1021 strongly induced autophagosome formation in macrophages after 1 and 2 h of incubation, as determined by an increased number of LC3-positive puncta (Fig. 4a). In addition, ATB1021 significantly enhanced the LPS-mediated formation of LC3 puncta in BMDMs (Fig. 4a, b), suggesting that ATB1021 upregulates LPS-induced autophagy. Moreover, ATB1021 increased the protein levels of LC3II/LC3I in BMDMs after 30 min; the effect persisted for 24 h (Fig. 4c). The p62 protein level was significantly increased in BMDMs after 3 h and gradually decreased after 18 h (Fig. 4c), suggesting the activation of autophagic flux by ATB1021.

p62 ligand-induced autophagy was further assessed by transmission electron microscopy-based enumeration of autophagosomes. Autophagosomes and autolysosomes were evident in BMDMs that had been treated with ATB1021 for 6 h (Fig. 4d). The numbers of ATB1021-induced autophagosomes were significantly increased by LPS (Fig. 4d, e). Furthermore, ATB1021-induced autophagic flux was assessed by incubation with the autophagy inhibitor BafA1^39^. ATB1021 further increased the protein ratio of LC3-II/LC3-I induced by BafA1 in macrophages in the presence and absence of LPS (Fig. 4f), indicating that p62 ligand significantly increases autophagic flux. Consistent with a previous report^35^, the mammalian target of rapamycin (mTOR; a master regulator of several signaling pathways, including autophagy) was not modulated by ATB1021 in macrophages (Supplementary Fig. 4). However, ATB1021 markedly inhibited LPS-induced phosphorylation of Akt in macrophages (Supplementary Fig. 4). Therefore, p62 ligand triggers autophagic flux in macrophages and significantly augments autophagy in LPS-treated conditions.

### A p62 ligand activates mitophagy in macrophages

**Fig. 5:**
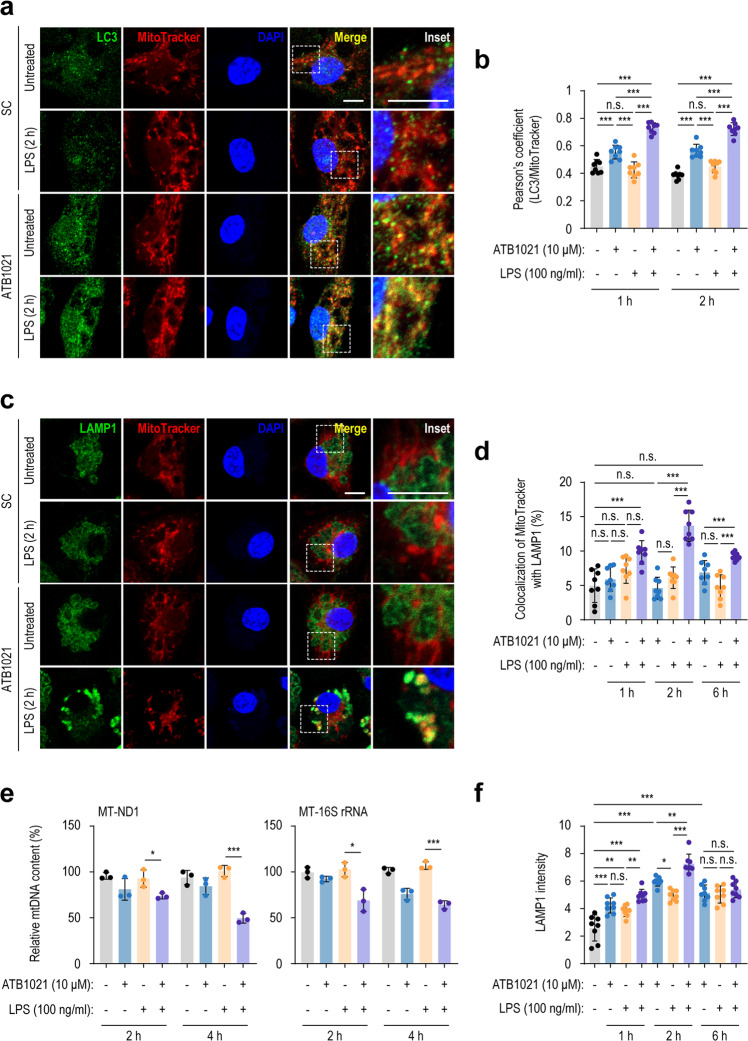
A p62 ligand activates mitophagy in macrophages. **a** PMs treated with vehicle or ATB1021 (10 μM) for 1 h and stimulated with LPS for 1 or 2 h were stained with anti-LC3 antibody (green), MitoTracker (red), and DAPI (nuclei; blue). Representative images (scale bar, 2 μm) are shown. The inset shows the cropped part of the merged images (scale bar, 2 μm). **b** Pearson’s correlation coefficient of LC3 and MitoTracker colocalization. **c**, **d** PMs treated for 1, 2, or 6 h as in (**a**) were stained with anti-LAMP1 antibody (green), MitoTracker (red), and DAPI (blue). Representative images obtained after 2 h of LPS stimulation (**c**) and quantitative analysis of the colocalization of MitoTracker with LAMP1 after 1, 2, or 6 h of LPS stimulation (**d**). Representative images with a scale bar of 2 μm are shown. The inset shows the cropped part of the merged images (scale bar, 2 μm). **e** PMs were pretreated with ATB1021 (10 μM) for 1 h and stimulated with LPS (100 ng/ml) for the indicated times, and then total DNA was extracted to quantify the relative mtDNA content of MT-ND1 and MT-16S rRNA. **f** Lysosomal intensity calculated based on the images in (**c**). Data are shown as the means ± SDs (**b**, **d**–**f**). Statistical significance was determined by one-way ANOVA with Tukey’s multiple comparison test (**b**, **d**, and **f**) or two-tailed Student’s *t* test (**e**). n.s. not significant, *p < 0.05, **p < 0.01, ***p < 0.001.

We showed that ATB1021, a synthetic ligand for p62 oligomerization, is involved in the activation of selective autophagy, but its role in mitophagy is unknown in the context of LPS stimulation. We thus assessed ATB1021-mediated mitophagy activation by enumerating mitophagosomes (MitoTracker^+^LC3^+^) and mitochondrial autolysosomes (MitoTracker^+^LAMP1^+^) in either unstimulated or LPS-stimulated PMs. Mitochondrial colocalization with LC3^+^ autophagosomes was significantly increased by ATB1021 alone or in LPS/ATB1021-treated PMs compared with LPS alone after 1 h or 2 h (Fig. 5a, b). In addition, the colocalization of mitochondria and LAMP1 in PMs was significantly enhanced by the combined treatment with ATB1021/LPS after 2–6 h; the effect peaked at 2 h of treatment (Fig. 5c, d). The formation of mitochondrial autolysosomes was not increased by ATB1021 alone compared with unstimulated conditions (Fig. 5d), indicating that ATB1021 per se does not trigger mitophagic flux in macrophages. The induction of mitophagy is further monitored by qPCR analysis of specific mitochondrial genes, such as MT-ND1 (mitochondrially encoded NADH dehydrogenase 1) and MT-16S rRNA, which can represent mitochondrial DNA (mtDNA) copy number per cell^39,40^. As shown in Fig. 5e, ATB1021 treatment significantly decreased mtDNA contents in LPS-stimulated BMDMs when compared to those with LPS treatment alone. Moreover, ATB1021 substantially increased the lysosomal intensities in LPS-stimulated PMs (Fig. 5f), suggesting that p62 ligand enhances lysosomal biogenesis during inflammation. These data strongly suggest that p62 ligand significantly induces the upregulation of mitophagy and lysosomal biogenesis in LPS-treated macrophages.

### p62 is required for ATB1021-mediated mitophagy activation, the suppression of LPS-induced proinflammatory responses and mtROS generation

**Fig. 6:**
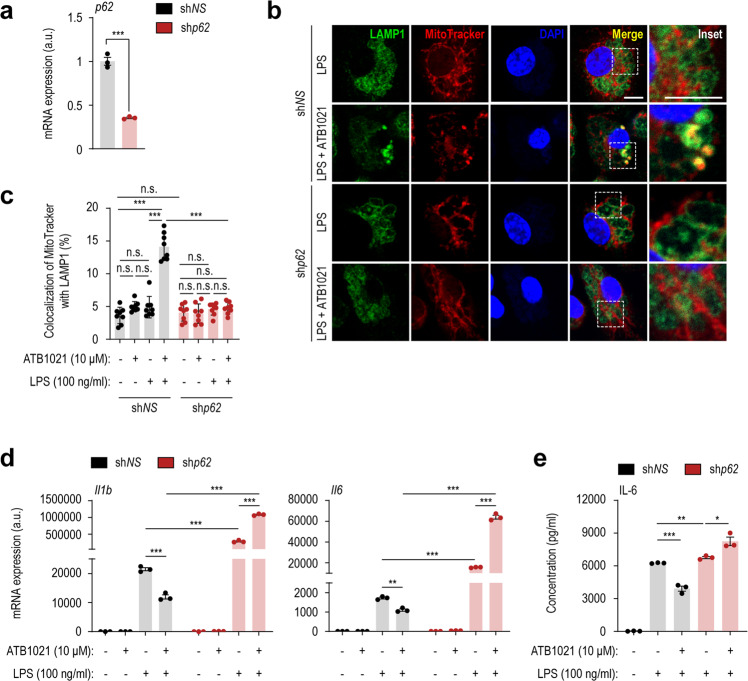
p62 ligand-mediated mitophagy activation and inhibition of LPS-induced inflammatory responses in macrophages is dependent on p62. **a** *p62* mRNA level in macrophages transduced with control (sh*NS*) or *p62*-specific shRNA (sh*p62*) (*n* = 3). **b** Control or *p62*-silenced PMs were treated with vehicle or ATB1021 (10 μM) for 1 h and stimulated with LPS (100 ng/mL) for 2 h; they were then stained with anti-LAMP1 antibody (green), MitoTracker (red), and DAPI (blue). Representative images are shown. Scale bar, 2 μm. The inset shows the cropped part of the merged images (scale bar, 2 μm). **c**, **d** sh*NS-* or sh*p62-*transduced macrophages were treated with vehicle or ATB1021 (10 μM) for 1 h and stimulated with LPS (100 ng/mL) for 6 h prior to quantitative PCR analysis of *Il1b* and *Il6* (**d**) or 18 h prior to ELISA analysis of IL-6 in the supernatant (**e**) (*n* = 3). Means ± SDs are shown (**a**, **c**–**e**). Two-tailed Student’s *t* test (**a**) or one-way ANOVA with Tukey’s multiple comparison test (**c**–**e**) was used to determine statistical significance. n.s., not significant, *p < 0.05, **p < 0.01, ***p < 0.001.

We recently showed that p62 is essential for the function of p62 ligands upon xenophagy activation^35^. To examine whether p62 is involved in ATB1021-mediated mitophagy activation and the regulation of proinflammatory responses in response to LPS, we used a lentiviral shRNA targeting *p62* (sh*p62*) to silence *p62* expression (Fig. 6a). The combined treatment of BMDMs with LPS and ATB1021 significantly increased the colocalization of mitochondria with lysosomes (Fig. 6b, c). The silencing of *p62* by sh*p62* considerably reduced the colocalization of mitochondria and lysosomes upregulated by the combined treatment with LPS and ATB1021 compared to those that had been transduced with the nonspecific control shRNA (sh*NS*) (Fig. 6b, c).

We next assessed the effect of *p62* silencing on the ATB1021-mediated suppression of proinflammatory cytokine production in response to LPS stimulation in PMs. In sh*NS*-transduced control cells, treatment of BMDMs with ATB1021 significantly suppressed the LPS-induced mRNA levels of *Il1b* and *Il6* after 3 and 6 h (Fig. 6d and Supplementary Fig. 5a, b), as well as the protein level of IL-6 after 18 h (Fig. 6e). Blockade of p62 by sh*p62* significantly increased the LPS-induced mRNA levels of *Il1b* and *Il6*, as well as the protein level of IL-6, compared with those in the sh*NS*-transduced condition (Fig. 6d, e, and Supplementary Fig. 5a, b). Notably, the silencing of *p62* substantially upregulated inflammatory cytokine generation in BMDMs treated with either LPS alone or the combination of LPS plus ATB1021 (Fig. 6d, e, and Supplementary Fig. 5a, b).

**Fig. 7:**
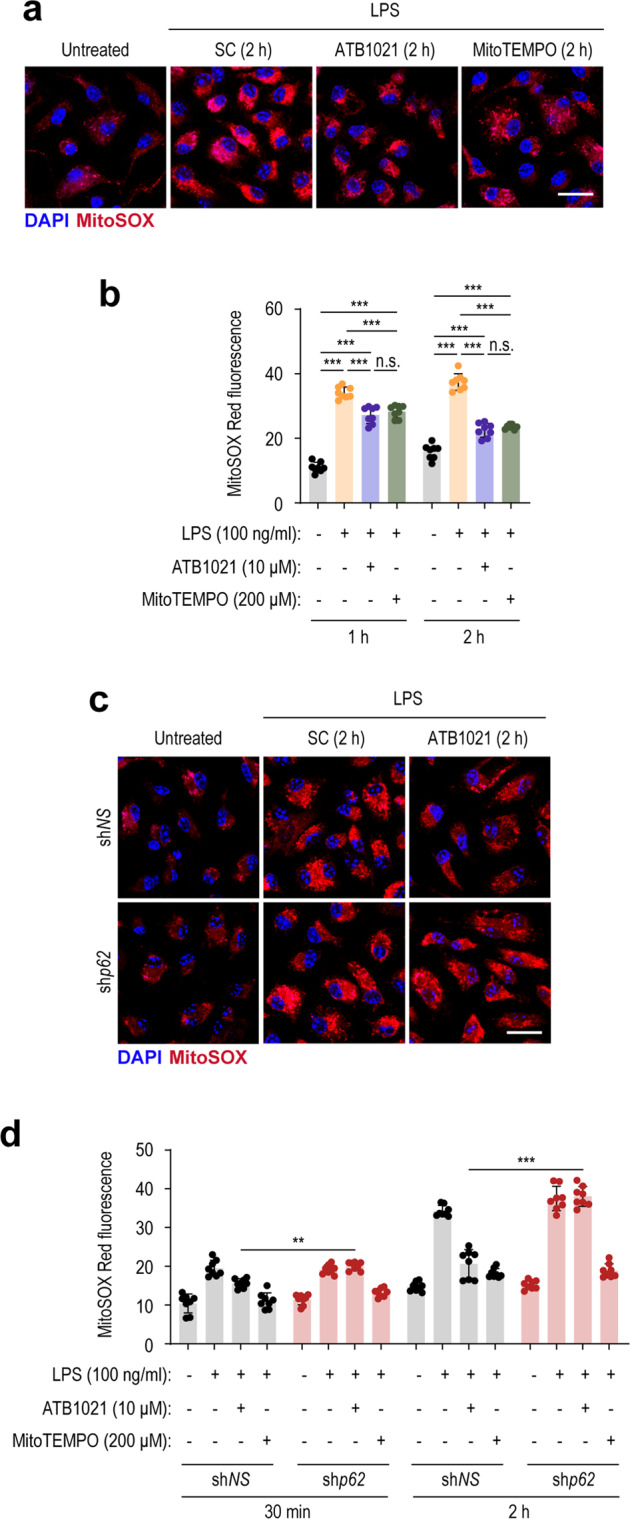
A p62 ligand inhibits LPS-induced mtROS production in a p62-dependent manner. **a**, **b** PMs were pretreated with vehicle, ATB1021 (10 μM), or MitoTEMPO (200 μM) and then stimulated with LPS (100 ng/mL) for 1 h or 2 h; next, they were stained with MitoSOX (red) or DAPI (blue). Representative images (**a**) and MitoSOX fluorescence intensities (**b**). Scale bar, 10 μm. **c**, **d** PMs transduced with sh*NS* or sh*p62* were pretreated with vehicle, ATB1021 (10 μM), or MitoTEMPO (200 μM) and then stimulated with LPS (100 ng/mL) for 30 min or 2 h; next, they were stained with MitoSOX (red) or DAPI (blue). Representative images (**c**) and MitoSOX Red fluorescence intensities (**d**). Scale bar, 10 μm. Means ± SDs are shown (**b**, **d**). One-way ANOVA with Tukey’s multiple comparison test (**b**, **d**) was used to determine statistical significance. n.s., not significant, **p < 0.01, ***p < 0.001.

Because macrophage inflammatory responses can be activated by mitochondrial reactive oxygen species (mtROS)^41^, we examined the effect of ATB1021 on LPS-induced mtROS production and whether this effect is dependent on p62. MitoSOX staining showed that LPS-induced mtROS production was significantly suppressed by ATB1021 in PMs, similar to the effect of MitoTEMPO, a scavenger of mtROS (Fig. 7a, b). The silencing of *p62* by sh*p62* considerably increased mtROS production in PMs in response to the combined treatment with LPS and ATB1021 after 30 min or 2 h (Fig. 7c, d). However, MitoTEMPO treatment significantly reduced LPS-triggered mtROS generation, regardless of *p62* knockdown (Fig. 7d). These data suggest that ATB1021-mediated control of mitochondrial oxidative stress upon LPS stimulation is dependent on *p62* expression. Therefore, p62 is the primary target for the effects of ATB1021 on mitophagy activation and anti-inflammatory and antioxidative responses in macrophages during LPS stimulation.

### A p62 ligand downregulates aerobic glycolysis and lactate production in macrophages during inflammation

**Fig. 8:**
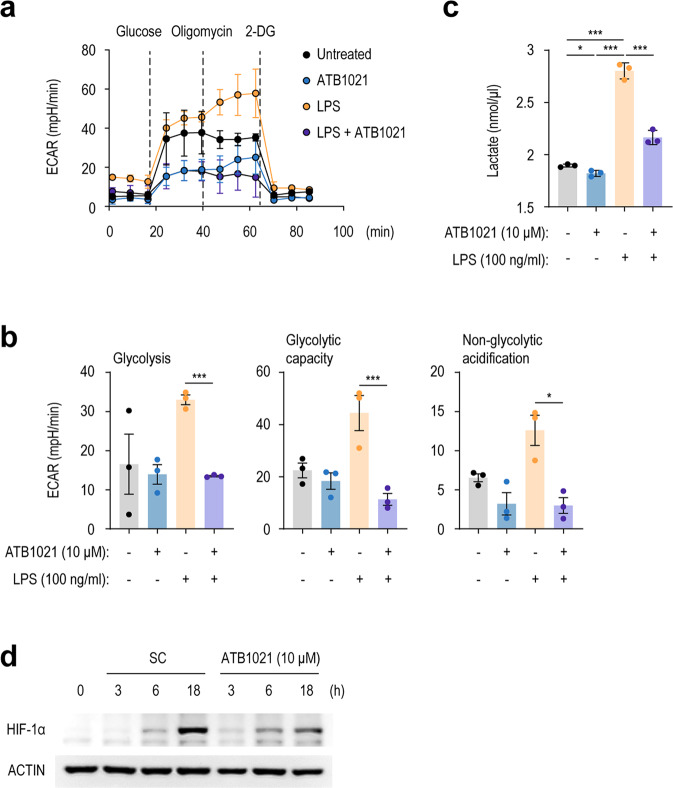
Inhibition of aerobic glycolysis and lactate production by a p62 ligand in LPS-stimulated macrophages. **a**, **b** ECAR profile (**a**) and representative ECAR parameters (**b**) in PMs pretreated with vehicle or ATB1021 (10 μM) for 1 h and stimulated with LPS for 6 h. **c** Lactate levels in the supernatants of PMs pretreated with vehicle or ATB1021 (10 μM) for 1 h and stimulated with LPS for 18 h (*n* = 3). **d** PMs were pretreated with vehicle or ATB1021 (10 μM) for 1 h and then stimulated with LPS for 3, 6, or 18 h; next, they were subjected to Western blotting analysis of HIF-1α. Means ± SDs are shown (**a**–**c**). Two-tailed Student’s *t* test (**b**, **c**) was used to determine statistical significance. ns; not significant, *p < 0.05, ***p < 0.001.

Immunometabolism is essential for regulating inflammation, and several immunomodulatory metabolites regulate the functional responses of immune cells^42–44^. The inhibition of glycolysis by targeting glycolytic enzymes or by metabolites can suppress inflammatory responses^45,46^. Therefore, we explored the modulating effect of a p62 ligand on immunometabolic responses to LPS by measuring aerobic glycolysis in macrophages. We monitored the ECAR using the Seahorse XF24 Analyzer and a glycolysis stress test. LPS-stimulated upregulation of aerobic glycolysis in PMs was significantly suppressed by ATB1021 (Fig. 8a, b). Notably, LPS-induced ECAR parameters, such as glycolysis, glycolytic capacity, and nonglycolytic acidification, were considerably suppressed by ATB1021 (Fig. 8b). Consistent with this finding, LPS-induced production of lactate (a product of glycolysis) was dramatically suppressed by ATB1021 (Fig. 8c). Furthermore, HIF-1α, a critical target during aerobic glycolysis and a regulator of immune responses^47^, was inhibited by ATB1021 in macrophages that had been stimulated with LPS (Fig. 8d). Therefore, the p62 ligand ATB1021 drives macrophage remodeling of immunometabolism to suppress aerobic glycolysis during inflammation.

## Discussion

Accumulating evidence implies that the autophagy receptor p62 functions as an N-recognin to connect the N-degron pathway, ubiquitin‒proteasome system, and autophagy process^32,34,48^. We recently showed that synthetic ligands to the p62 ZZ domain enhance reticulophagy and protein quality control in the endoplasmic reticulum^32,34^. Additionally, p62 ligands activate xenophagy to enhance antimicrobial host defenses against intracellular bacteria during infection^35^. Despite this, it remains undefined mainly whether p62 ligands regulate inflammatory responses in the context of sepsis, a severe inflammatory disorder, and how they could be controlled. Herein, we found that the p62 ligand ATB1021 played protective roles in septic shock and experimental sepsis and strongly augmented LPS-induced autophagy and mitophagy in macrophages. Mechanistically, p62 was required for p62 ligand-mediated mitophagy, anti-inflammation, and the maintenance of mitochondrial homeostasis during inflammation. Moreover, a p62 ligand suppressed LPS-induced aerobic glycolysis and lactate production in macrophages. These data highlight the potential therapeutic application of the p62 ligand for sepsis and septic shock through orchestrating mitophagy and immunometabolic remodeling during inflammation.

There are several strategies for controlling sepsis, including administering corticosteroids^49,50^. However, corticosteroids might increase the mortality risk in patients with infection or septic shock^51^. In addition, the use of corticosteroids might increase the risk of hyperglycemia^52^. We previously showed that p62 ligands promote antimicrobial responses to pathogens such as *Salmonella enterica* serovar Typhimurium, *Mycobacterium tuberculosis*, and *Escherichia coli*^35^. Combined with the current findings that p62 ligands are effective in polymicrobial sepsis and preventing organ damage, the available data strongly suggest that p62 ligands are a beneficial therapeutic approach in the context of sepsis and septic shock caused by various pathogenic stimuli.

Notably, p62 ligands showed potent inhibitory effects on the production of IL-1β, IL-18, and IL-6, demonstrating their ability to reduce inflammatory injury in response to various innate immune stimuli. Indeed, a clinical trial showed that anakinra, an IL-1 inhibitor, reduced mortality and the length of hospitalization among patients with coronavirus disease 2019 who are susceptible to respiratory failure^53^. Another trial with anakinra revealed the suppression of circulating IL-6 levels^54^. Importantly, we found that ATB1021 attenuates the level of *Il18*, which is associated with poor clinical outcomes in severe inflammatory and septic disease^55–57^. Thus, our data strongly suggest that p62 ligands can be beneficial in treating inflammatory diseases in critically ill patients with pathological inflammation. In addition, p62 ligands significantly suppressed the expression of chemokines such as *Ccl2*, *Cxcl2*, and *Cxcl5*, which are associated with neutrophil recruitment. Neutrophils are the principal cells responsible for the enhanced inflammatory response during sepsis-associated ALI^58^. Because ATB1021 reduced neutrophil infiltration in our model of experimental sepsis (Supplementary Fig. 1e), a p62 ligand may be beneficial in preventing sepsis-related cytokine storms and ALI. Future clinical trials will clarify the effects of p62 ligands aiding the prevention and treatment of severe sepsis and pathological inflammatory responses in critically ill septic patients.

The p62 ligand ATB1021 strongly induced autophagy in unstimulated macrophages and amplified LPS-induced autophagy. LPS is a TLR4 ligand that triggers autophagy in macrophages through a Toll/IL-1 receptor domain-containing adapter-inducing interferon-β-dependent pathway^59,60^. Our findings with the p62 ligand-mediated augmentation of autophagy may contribute to preventing LPS-induced hyperactivation of inflammatory responses. In this study, we demonstrate the unique function of p62 ligands upon the activation of mitophagy in macrophages in the context of LPS-induced inflammation. Mitophagy activation is critically required for preserving mitochondrial function, while mitochondrial damage impedes immune function and cell survival during inflammation^61,62^. Notably, p62 played a significant role in the p62 ligand-mediated anti-inflammation, antioxidative effects, and mitophagy activation in macrophages during inflammation. Indeed, mitophagy is essential for maintaining immune homeostasis and immunoregulation in response to various stresses^61,63^. Thus, the current data strongly suggest that the p62 ligand-mediated activation of mitophagy plays a critical role in controlling inflammation by eliminating damaged mitochondria and scavenging mtROS in response to LPS stimulation.

Our data demonstrate that ATB1021 modulated LPS-induced aerobic glycolysis in macrophages. In response to proinflammatory signals, various immune cells (including myeloid cells) exhibit upregulation of aerobic glycolysis^64,65^. In classically activated macrophages, the broken Krebs cycle leads to the accumulation of metabolites (e.g., succinate), thereby enhancing IL-1β expression via reactive oxygen species-dependent stabilization of HIF-1α^65,66^. In addition, the Akt/ERK pathway contributes to the translational activation of HIF-1α^67^. Although it is unclear, p62 ligand-mediated suppression of HIF-1α expression is presumably the result of inhibition of Akt/ERK (Supplementary Fig. 4 and Fig. 8d). Indeed, mitophagy activation is critical for the regulation of mitochondrial dynamics and metabolic shifts in response to cell type and demand^68^. Therefore, p62 ligand-induced mitophagy activation may be associated with macrophage-specific immunometabolic remodeling leading to an anti-inflammatory phenotype during inflammation. Future studies should clarify the role of the p62 ligand in the association between mitophagy and immunometabolism during inflammation.

In conclusion, our data demonstrate that p62-targeting N-degron mimetics are promising candidates for preventing and treating sepsis and septic shock. The p62 ligand suppressed the production of inflammatory cytokines in vitro and in vivo, activated mitophagy, and maintained mitochondrial homeostasis via p62. Moreover, the p62 ligand suppressed aerobic glycolysis by inhibiting HIF-1α in response to LPS. Because anti-IL-1β/IL-6 strategies have therapeutic potential for human diseases (e.g., autoimmune and autoinflammatory conditions, as well as coronavirus disease 2019-related sepsis), p62 ligands should be developed as therapeutic agents for a variety of immune-related diseases, particularly systemic inflammation.

## Supplementary information

Supplementary Figures

## Data Availability

The datasets used and/or analyzed during the current study are available from the corresponding author upon reasonable request.
