# Supplementary material for: Chemical mimetics of the N-degron pathway alleviate systemic inflammation by activating mitophagy and immunometabolic remodeling
Source: Exp Mol Med. 2023 Feb 1;55(2):333–46. doi: 10.1038/s12276-023-00929-x (PMC9981610; doi:10.1038/s12276-023-00929-x)
Supplement: Supplementary file 1 — Supplementary Figures [file 12276_2023_929_MOESM1_ESM.docx]

**Supplementary information for**

‘Chemical mimetics of the N-degron pathway alleviate systemic inflammation by activating mitophagy and immunometabolic remodeling’

**
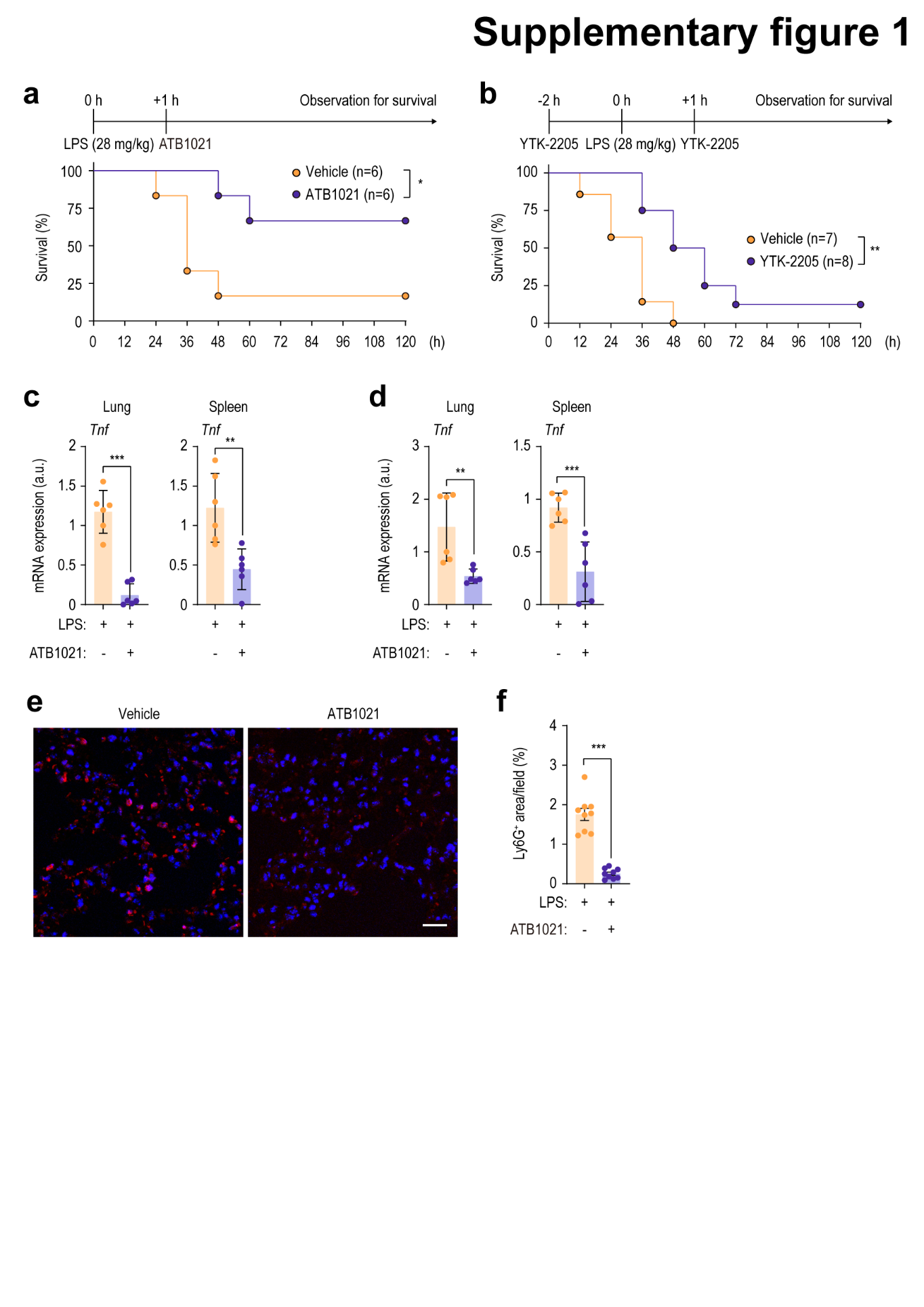

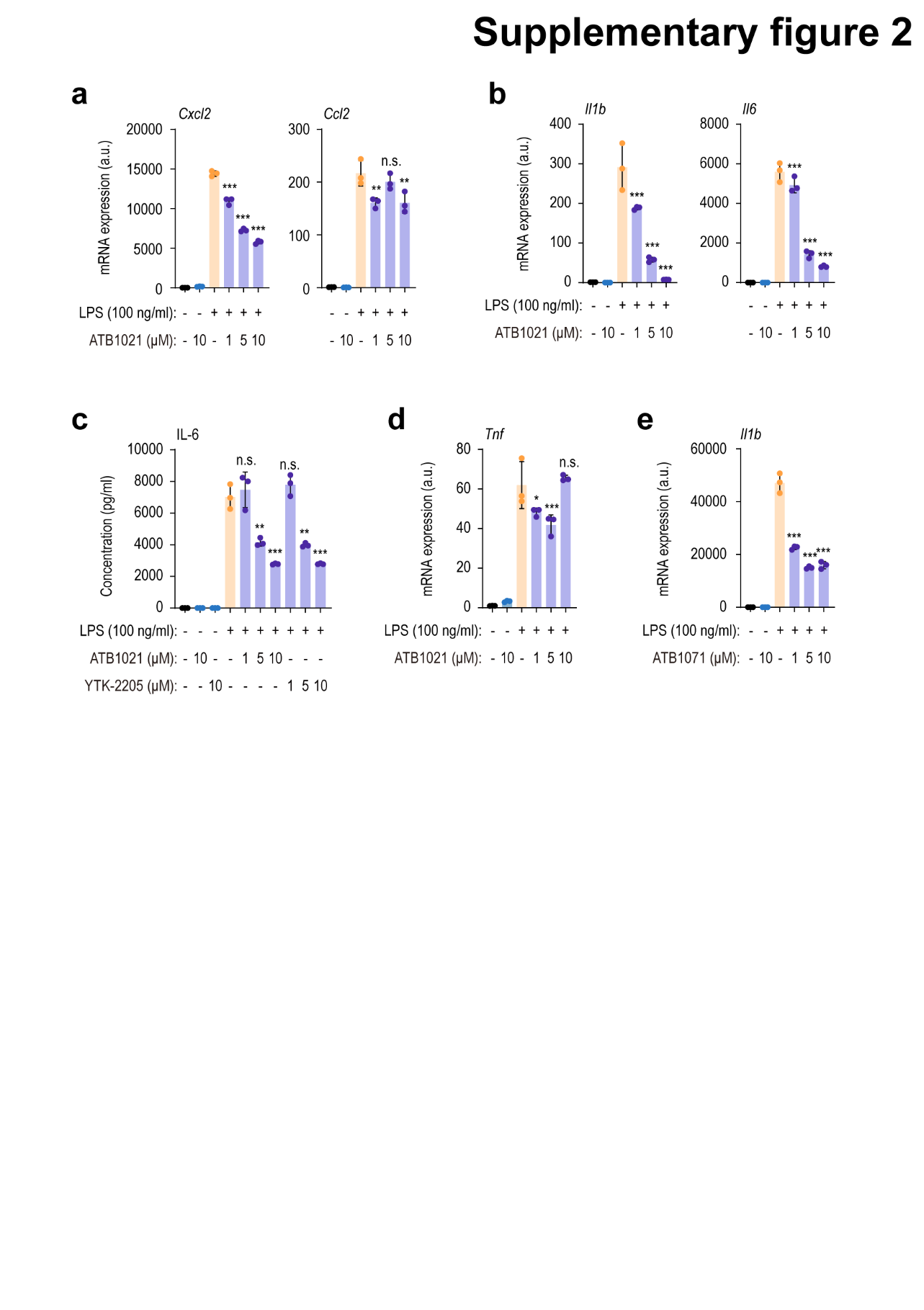
**

**
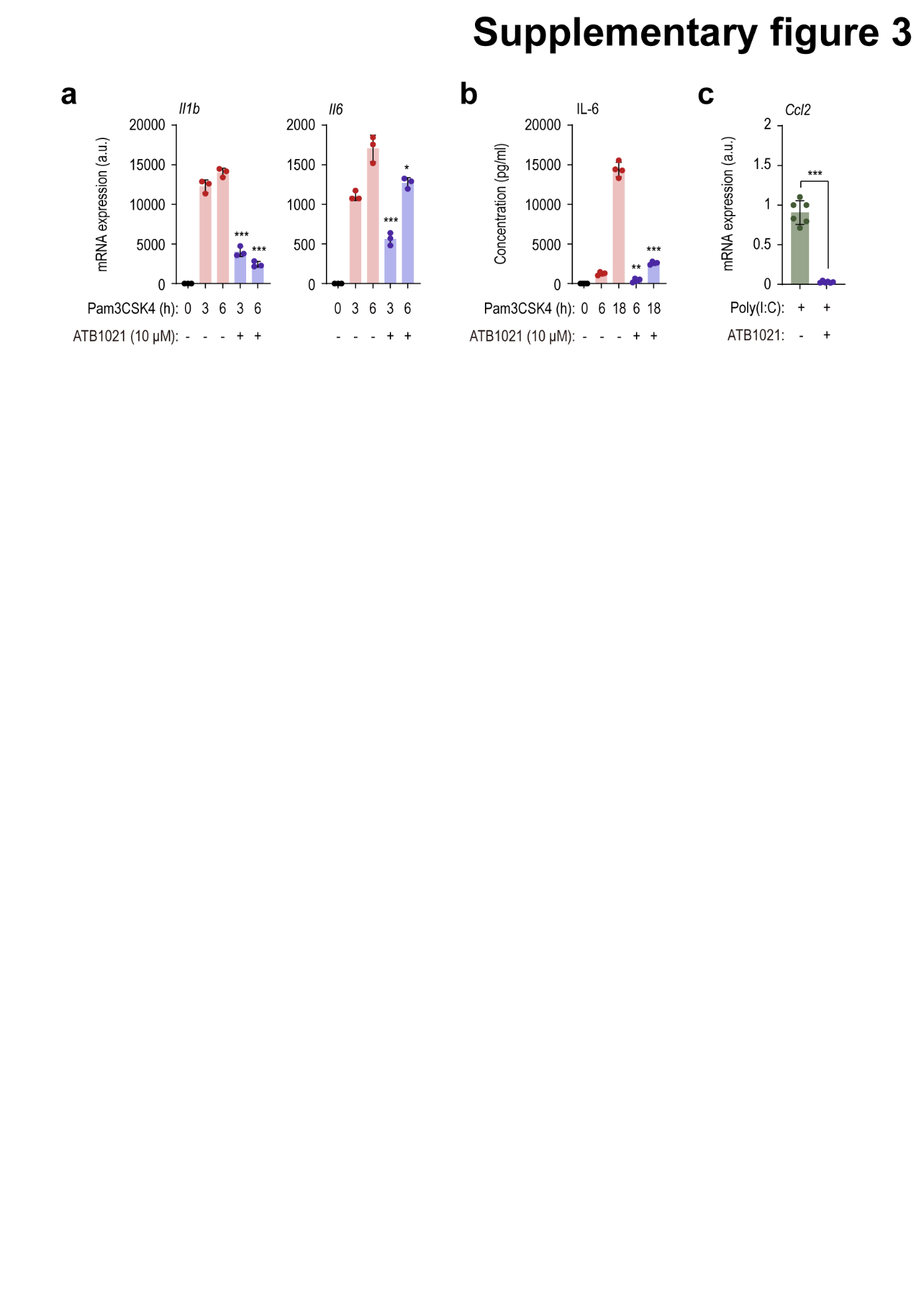
**

**
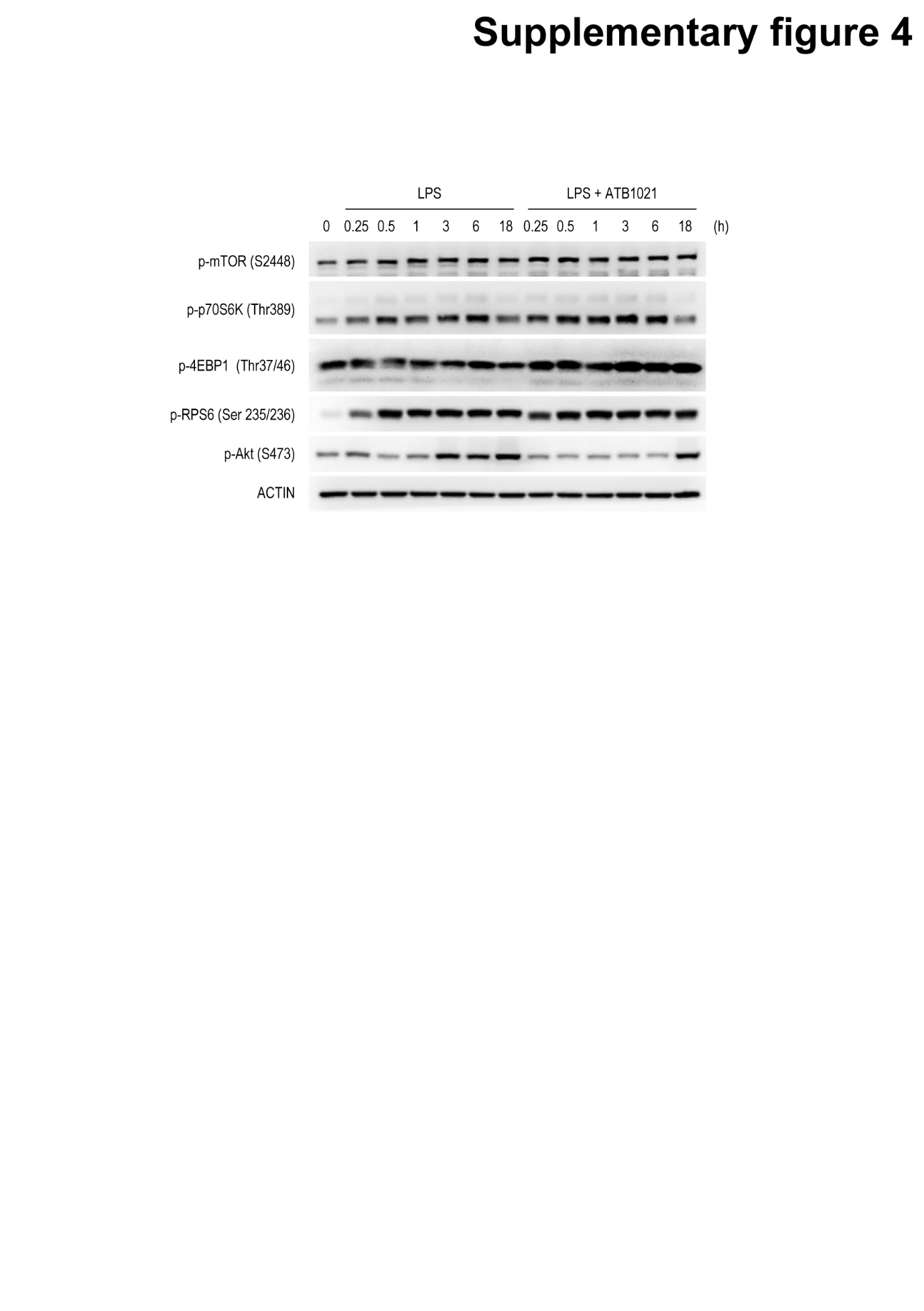
**

**
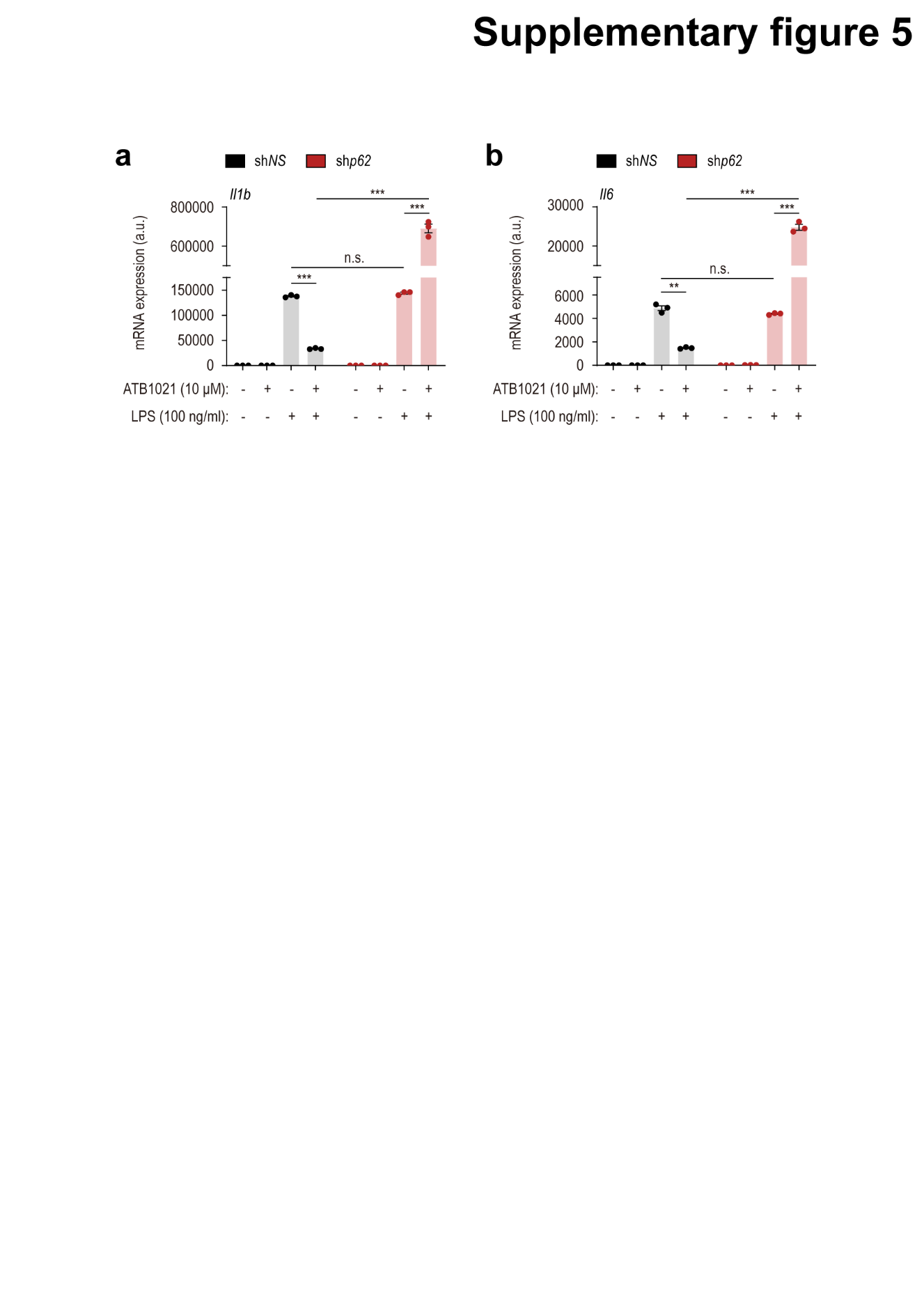
**

**Supplementary Figure Legends**

**Supplementary Fig. 1. Anti-inflammatory effects of p62 ligands *in vivo.* a**, **b** Survival of mice for 120 h after treatment with vehicle or ATB1021 (20 mg/kg) 1 h after (**a**) or YTK-2205 (20 mg/kg) for 2 h before and 1 h after (**b**) LPS injection (28 mg/kg) (n=6-8 per each group). **c**, **d** mRNA level of Tnf in lung and spleen tissues from LPS-injected mice treated with vehicle or ATB1021 (for 1 and 2 h, **c** and **d**, respectively; n=6). **e**, **f** Lung tissues from Fig. 1h were stained with anti-Ly6G antibody (red) and DAPI (blue). Representative images (**e**) and quantification of Ly6G+ per field (**f**). Data are means ± SEM (**c**, **d**, **f**). Log-rank (Mantel-Cox) test (**a**, **b**) or two-tailed Student’s *t* test (**c**, **d**, **f**) was used to determine statistical significance. n.s.; not significant, *p < 0.05, **p < 0.01, ***p < 0.001.

**Supplementary Fig. 2. Inhibition of LPS-induced proinflammatory cytokine and chemokine production by p62 ligands. a** PMs pretreated with ATB1021 were stimulated with LPS (100 ng/mL) for 6 h, then analyzed to determine mRNA levels of *Cxcl2* and *Ccl2* (n=3). **b**, **c**, **d** mRNA levels of *Il1b* and *Il6* (**b**) and the protein level of IL-6 (**c**) and the mRNA level of *Tnf* (**d**) were analyzed in BMDMs (**b**) and in PMs pretreated with ATB1021 (**b**, **c**, **d**) or YTK-2205 (**c**) and stimulated with LPS for 6 h (mRNA) or 18 h (protein) (n=3). **e** mRNA level of *Il1b* in PMs treated with ATB1071 and stimulated with LPS for 6 h (n=3). Mean ± SD (**a**-**e**) are shown. One-way ANOVA with Tukey’s multiple comparison test (**a**, **b**, **d**) or two-tailed Student's *t* tests (**c**, **e**) were used to determine statistical significance. n.s.; not significant; **p* < 0.05, ***p* < 0.01, ****p* < 0.001.

**Supplementary Fig. 3.** **Inhibition of innate immune stimuli by a p62 ligand. a, b** PMs were pretreated with vehicle or ATB1021 (10 μM) for 1 h and stimulated with Pam3CSK4 (100 ng/mL), then analyzed to determine the mRNA levels of *Il1b* and *Il6* (**a**) and the protein level of IL-6 in the supernatant (**b**). **c** mRNA levels of *Ccl2* in lung tissue as in Fig. **3e**. Mean ± SD are shown. Two-tailed Student’s *t* test was used to determine statistical significance. n.s.; not significant, **p* < 0.05, ***p* < 0.01, ****p* < 0.001.

**Supplementary Fig. 4. Effects of a p62 ligand on mTOR signaling in LPS-stimulated macrophages.** PMs were pretreated with vehicle or ATB1021 (10 μM) for 1 h and stimulated with LPS (100 ng/mL), then subjected to Western blotting to assess the phosphorylation of Akt/mTOR-related signaling factors.

**Supplementary Fig. 5. p62 ligand-mediated inhibition of LPS-induced inflammatory responses in macrophages is p62-dependent. a**, **b** mRNA levels of *Il1b* (**a**) or *Il6* (**b**) in *p62*-silenced PMs (Fig. 6c) stimulated with LPS for 6 h. Data are means ± SD. Two-tailed Student’s *t* test was used to determine statistical significance. n.s.; not significant, ***p* < 0.01, ****p* < 0.001.**Supplementary Table 1.** Primers used in this study.

| Genes (mouse) | Primer | Sequences |
| --- | --- | --- |
| *Ccl2* | Forward  Reverse | 5′-TGACCCCAAGAAGGAATGGG-3′  5′-ACCTTAGGGCAGATGCAGTT-3′ |
| *Cxcl2* | Forward  Reverse | 5′-CCCTGCCAAGGGTTGACTTC-3′  5′-GCAAACTTTTTGACCGCCCT-3′ |
| *Cxcl5* | Forward  Reverse | 5′-CCGCTGGCATTTCTGTTGCTGT-3′  5′-CAGGGATCACCTCCAAATTAGCG-3′ |
| *Cxcl9* | Forward  Reverse | 5′-AACGTTGTCCACCTCCCTTC-3′  5′-CACAGGCTTTGGCTAGTCGT-3′ |
| *Cxcl10* | Forward  Reverse | 5′-CCAAGTGCTGCCGTCATTTT-3′  5′-CTCAACACGTGGGCAGGATA-3′ |
| *Il18* | Forward  Reverse | 5′-TCAGACAACTTTGGCCGACT-3′  5′-GGTGGATCCATTTCCACTTTGA-3′ |
| *Il1b* | Forward  Reverse | 5′-TACGGACCCCAAAAGATGA-3′  5′-TGCTGCTGCGAGATTTGAAG-3′ |
| *Il6* | Forward  Reverse | 5′-TACCACTTCACAAGTCGGAGGC-3′  5′-CTGCAAGTGCATCATCGTTGTTC-3′ |
| *Tnf* | Forward  Reverse | 5′-ACGGCATGGATCTCAAAGAC-3′  5′-AGATAGCAAATCGGCTGACG-3′ |
| *Gapdh* | Forward  Reverse | 5′-AAGATGGTGATGGGCTTCCCG-3′  5′-TGGCAAAGTGGAGATTGTTGCC-3′ |
